# Contractile force measurement of human induced pluripotent stem cell-derived cardiac cell sheet-tissue

**DOI:** 10.1371/journal.pone.0198026

**Published:** 2018-05-23

**Authors:** Daisuke Sasaki, Katsuhisa Matsuura, Hiroyoshi Seta, Yuji Haraguchi, Teruo Okano, Tatsuya Shimizu

**Affiliations:** 1 Institute of Advanced Biomedical Engineering and Science, Tokyo Women’s Medical University, Tokyo, Japan; 2 Department of Cardiovascular Surgery, Tokyo Women’s Medical University, Tokyo, Japan; University of Tampere, FINLAND

## Abstract

We have developed our original tissue engineering technology “cell sheet engineering” utilizing temperature-responsive culture dishes. The cells are confluently grown on a temperature-responsive culture dish and can be harvested as a cell sheet by lowering temperature without enzymatic digestion. Cell sheets are high-cell-density tissues similar to actual living tissues, maintaining their structure and function. Based on this “cell sheet engineering”, we are trying to create functional cardiac tissues from human induced pluripotent stem cells, for regenerative therapy and *in vitro* drug testing. Toward this purpose, it is necessary to evaluate the contractility of engineered cardiac cell sheets. Therefore, in the present study, we developed a contractile force measurement system and evaluated the contractility of human iPSC-derived cardiac cell sheet-tissues. By attaching the cardiac cell sheets on fibrin gel sheets, we created dynamically beating cardiac cell sheet-tissues. They were mounted to the force measurement system and the contractile force was measured stably and clearly. The absolute values of contractile force were around 1 mN, and the mean force value per cross-sectional area was 3.3 mN/mm^2^. These values are equivalent to or larger than many previously reported values, indicating the functionality of our engineered cardiac cell sheets. We also confirmed that both the contractile force and beating rate were significantly increased by the administration of adrenaline, which are the physiologically relevant responses for cardiac tissues. In conclusion, the force measurement system developed in the present study is valuable for the evaluation of engineered cardiac cell sheet-tissues, and for *in vitro* drug testing as well.

## Introduction

Recent advances in tissue engineering are greatly promoting its application to regenerative therapies, *in vitro* drug testing, and pathological investigations. One of the most widespread methodologies in tissue engineering is to mix cells with a biocompatible scaffold of natural and/or synthetic polymers such as collagen gel, poly(lactide-co-glycolide), and so on [1, 2]. As an alternative approach, we have developed our original scaffold-free tissue engineering methodology, “cell sheet engineering”, by utilizing temperature-responsive culture dishes [3–6]. On the surface of these dishes, a temperature-responsive polymer, poly(*N*-isopropylacrylamide) (PIPAAm), is grafted covalently with nanometer-order thickness. The surface wettability changes sharply across 32°C. At 37°C, where cells are cultivated, the surface is relatively hydrophobic and allows for cell attachment. When the temperature is lowered below 32°C, the surface becomes highly hydrophilic and prevents cell attachment. Therefore, the cells confluently cultured on this surface can be harvested as an intact cell sheet only by lowering temperature. Because of the unnecessity of enzymatic digestion, the cell sheet maintains its membrane structure with intact proteins, intercellular connections similar to actual living tissues, and thus its biological functions. We can also create thicker cell sheet-tissues by stacking the cell sheets [7, 8]. Several clinical studies and trials of regenerative therapies based on the transplantation of cell sheet-tissues have been started in the fields of cardiovascular surgery [9], ophthalmology [10], gastrointestinal surgery [11], and oral surgery [12]. These studies demonstrated the therapeutic effectiveness associated with the high functionality of cell sheet-tissues as transplants.

As for cardiac regenerative therapy, the autologous skeletal myoblast sheets are presently transplanted to the failing heart to recover the damaged cardiac tissues mainly by their paracrine effects [9]. Recent drastic progress in the field of pluripotent stem cells (embryonic stem cells (ESCs) and induced pluripotent stem cells (iPSCs)) enables the preparation of adequate amounts of human cardiomyocytes to create cardiac tissues [13–15]. These advancements are opening the door for next-generation cardiac regenerative therapies based on the transplantation of engineered beating cardiac tissues that can directly assist the blood circulation. Motivated by such an objective, we are trying to create beating cardiac tissues with powerful contractility as transplants for cardiac regenerative therapies using cell sheet engineering [16–23]. To achieve this purpose, the evaluation of the contractility of engineered cardiac cell sheet is indispensable. The contractility of cardiac cell sheets may be evaluated by several conventional methods, such as the quantification of contractile proteins by immunostaining, or the microscopic motion analysis of cardiac beating. However, the most direct and reliable index for the evaluation of contractility is of course the contractile force.

As for the method to measure the contractile force of engineered cardiac tissues, there are several preceding researches. Schaaf et al. [24] and Turnbull et al. [25] measured the contractile force of human ESC-derived engineered cardiac tissues, which were prepared as the mixture of cardiomyocytes and fibrin or collagen type I based gel. The cardiac tissues were mounted to flexible silicone posts, and the contractile force was analyzed by detecting the deflection of the posts. Tulloch et al. [26], Masumoto et al. [27], and Ruan et al. [28] measured the contractile force of human ESC- or iPSC-derived engineered cardiac tissues, which were prepared as the mixture of cardiomyocytes and collagen type I gel, by connecting them to a force transducer (Aurora Scientific, Aurora, ON, Canada). Zhang et al. [29] measured the contractile force of human ESC-derived engineered cardiac tissues, which were prepared as the mixture of cardiomyocytes and fibrin gel, by connecting them to an optical force transducer. In the present study, we developed a method and system to measure the contractile force of human iPSC-derived cardiac cell sheet-tissues. We also investigated the possibility of the developed system as an *in vitro* drug testing platform.

## Materials and methods

The animal experiments (S1 Fig) were performed according to the “Guidelines of Tokyo Women’s Medical University on Animal Use” under the approval of institutional ethical committee (approval number: 13–63).

### Human iPSC culture

We used human iPSC line 201B7 purchased from RIKEN (Tsukuba, Japan). In this iPSC line, the puromycin-resistance gene under the control of an α-myosin heavy chain promoter was transferred as previously described [30]. The undifferentiated iPSCs were cultured in Primate ES Cell Medium (ReproCELL, Yokohama, Japan) on mitomycin C-treated mouse embryonic fibroblasts (ReproCELL) in the presence of 5 ng/ml basic fibroblast growth factor (ReproCELL) at 37°C in a humidified atmosphere with 5% CO_2_. The iPSCs were passaged every 3–4 days by using CTK solution (ReproCELL).

### Cardiac differentiation of human iPSCs in a bioreactor system

Cardiac differentiation of iPSCs was induced with slight modifications to the procedure previously described [15]. Briefly, iPSC aggregates were harvested from culture dishes using CTK solution treatment. The aggregates were then cultured in a stirred bioreactor system (Bio Jr.8; Able, Tokyo, Japan) with mTeSR1 (STEMCELL Technologies, Vancouver, Canada) containing 10 μM Y27632 (Wako Pure Chemical Industries, Osaka, Japan) (Day 0). On the next day (Day 1), the culture medium was changed to mTeSR1 without Y27632. On Day 2, the culture medium was changed to StemPro34 medium (Thermo Fisher Scientific, Waltham, MA, USA) containing 50 μg/ml ascorbic acid (Sigma-Aldrich, St. Louis, MO, USA), 2 mM L-glutamine, and 400 μM 1-thioglycerol (Sigma-Aldrich). Additionally, 0.5 ng/ml BMP4 (R&D systems, Minneapolis, MN, USA) from Day 2 to Day 3, 10 ng/ml BMP4, 5 ng/ml bFGF, and 3 ng/mL Activin A (R&D systems) from Day 3 to Day 6, 4 μM IWR-1 (Wako Pure Chemical Industries) from Day 6 to Day 8, 5 ng/mL VEGF (R&D systems) and 10 ng/mL bFGF from Day 8 to Day 16, were added. The culture medium was changed to fresh medium on Day 3, 6, 8, 10, 12, and 14. The entire process was done in a stirred bioreactor system, in which the agitation rate was 40 rpm, the dissolved oxygen was maintained at 40% with air, oxygen, or nitrogen additions, the pH was maintained at 7.2 by CO_2_ addition, and the temperature was maintained at 37°C. On Day 15, differentiated iPSCs including cardiomyocytes were harvested from the bioreactor.

### Purification of human iPSC-derived cardiomyocytes

Human iPSC-derived cardiomyocytes were purified according to the method described previously [23]. The differentiated iPSCs containing cardiomyocytes harvested from the bioreactor were dissociated with 0.05% trypsin/EDTA treatment and seeded on culture dishes at 1.0–1.7 × 10^5^ cells/cm^2^ (Day 15). The cells were cultured in Medium A (defined as DMEM (D6429; Sigma-Aldrich) containing 10% FBS and Penicillin-Streptomycin (Sigma-Aldrich)) in a humidified incubator with 5% CO_2_ at 37°C (Panasonic Healthcare, Tokyo, Japan). On Day 20, 1.5 μg/ml puromycin (Sigma-Aldrich) was added for 24–36 hours to eliminate non-cardiomyocytes which did not express the puromycin-resistant gene. On the next day (Day 21), the cells were passaged with 0.05% trypsin/EDTA treatment and seeded on culture dishes at 1.0–1.7 × 10^5^ cells/cm^2^. The above process was repeated again. That is, 1.5 μg/ml puromycin was added again on Day 26, and on the next day (Day 27) the purified cardiomyocytes were harvested with 0.05% trypsin/EDTA treatment. The dissociated cells were suspended in a fresh medium, passed through a 70-μm nylon mesh cell strainer (Corning, Corning, NY, USA) to remove the cell aggregates, and used for cardiac cell sheet engineering. Through the purification process, the medium was changed to fresh medium on the day after cell seeding, and then every other day.

### Flow cytometry

The percentage of iPSC-derived cardiomyocytes after the purification process was analyzed by flow cytometry. The differentiated iPSCs were fixed with 4% paraformaldehyde for 10 minutes, and labeled with cardiac troponin t (cTnT) antibody (MS295-P1 clone 13–11, Thermo Fisher Scientific) in PBS containing 0.2% Nonidet P-40 (Nacalai Tesque, Kyoto, Japan) and 5% FBS. As isotype controls, the cells were labeled with Mouse IgG1 (X0931; Dako-Agilent, Santa Clara, CA, USA) instead of cTnT antibody. The cells were then labeled with Alexa Fluor 488 (A-11017; Thermo Fisher Scientific) and analyzed by Gallios flow cytometer (Beckman Coulter, Brea, CA, USA) and Kaluza analysis software (Beckman Coulter).

### Fibrin gel sheet preparation

**Fig 1:**
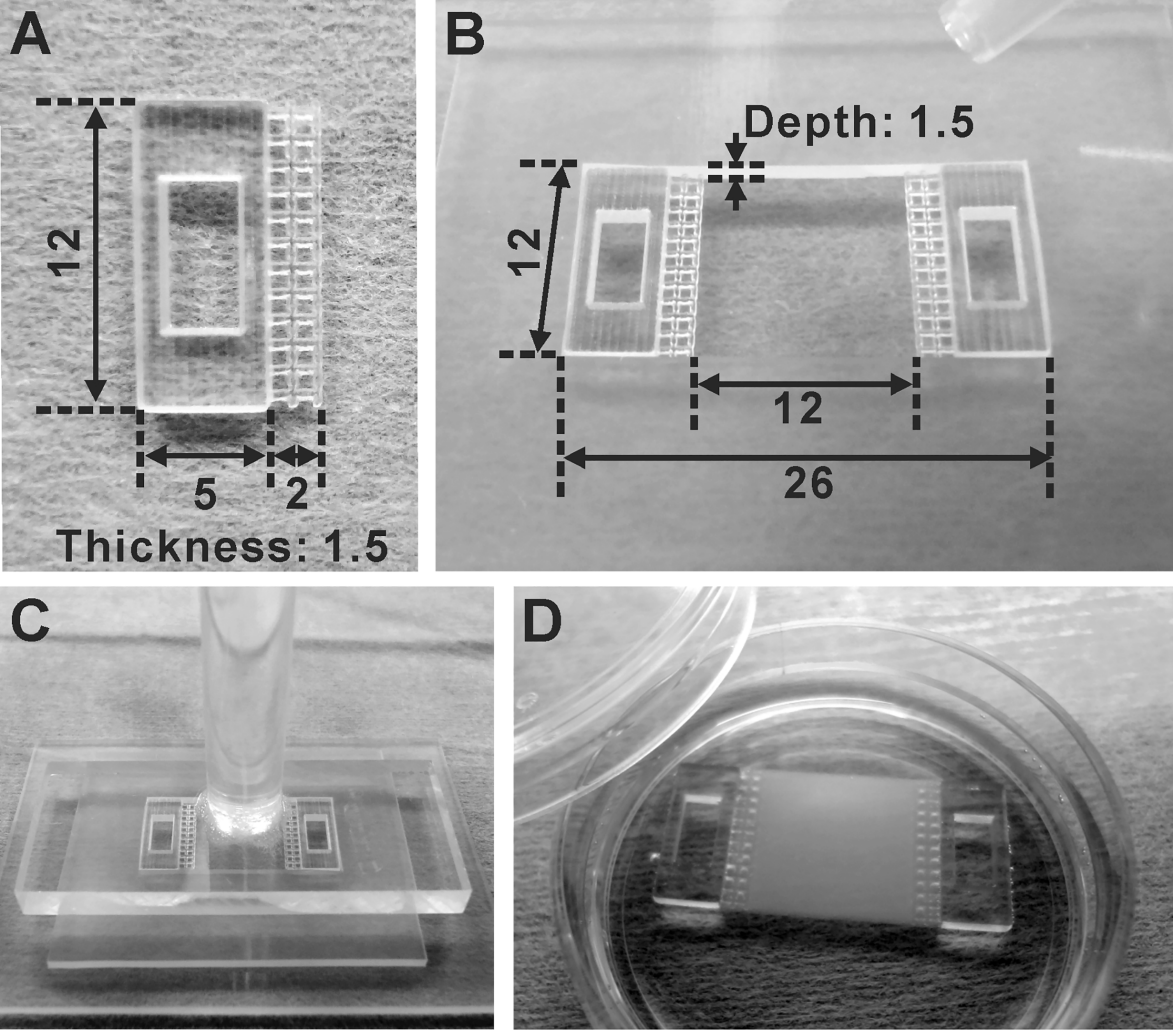
Preparation of fibrin gel sheets. (A) The handle made by a 3D printer to manipulate a fibrin gel sheet. The unit of measure of the numbers in the figure is mm. (B) The silicone mold in which two handles were put at both ends. The unit of measure of the numbers in the figure is mm. (C) Immediately after fibrin gel solution was poured in the silicone mold, the acrylic plate was put on it. The gel solution was clotted for 20 minutes at room temperature. (D) The prepared fibrin gel sheet.

Fibrin gel sheets were prepared as the bases of cardiac cell sheets for contractile force measurement. By using a 3D printer (Objet Eden350; Stratasys, Eden Prairie, MN, USA), 12 mm × 5 mm × 1.5 mm thick plastic plates with a 6 mm × 3 mm rectangular hole and a 12 mm × 2 mm mesh at one side were made of ultraviolet-curing resin (MED610; Stratasys) as shown in Fig 1A. The plates were used as handles to manipulate fibrin gel sheets. By using silicone sheets, 12 mm × 26 mm × 1.5 mm depth molds were prepared and two handles were put on both sides of each mold as shown in Fig 1B. Fibrinogen (Bolheal; Kaketsuken, Kumamoto, Japan), thrombin (Bolheal; Kaketsuken), and CaCl_2_ were dissolved in saline and mixed at the concentrations of 11.1 mg/ml, 0.5 units/ml, and 2 mM, respectively. Immediately after the mixing, the solution was poured into the silicone mold and an acrylic plate was placed on the mold (Fig 1C). The solution clotted to form fibrin gel within a few minutes. After 20 minutes, the fibrin gel sheet with handles was picked up from the mold and placed in a dish (Fig 1D). The fibrin gel sheets were immersed in Medium B (defined as Medium A containing additional 30 mM KCl and 500 KIU/ml aprotinin (Wako Pure Chemical Industries)) with 20 μg/ml fibronectin (Corning) for more than 2 hours at 37°C, and used for cell sheet transfer. All the tools used for fibrin gel sheet preparation were sterilized beforehand by washing with 70% ethanol and ultraviolet irradiation.

### Cardiac cell sheet-tissue engineering

**Fig 2:**
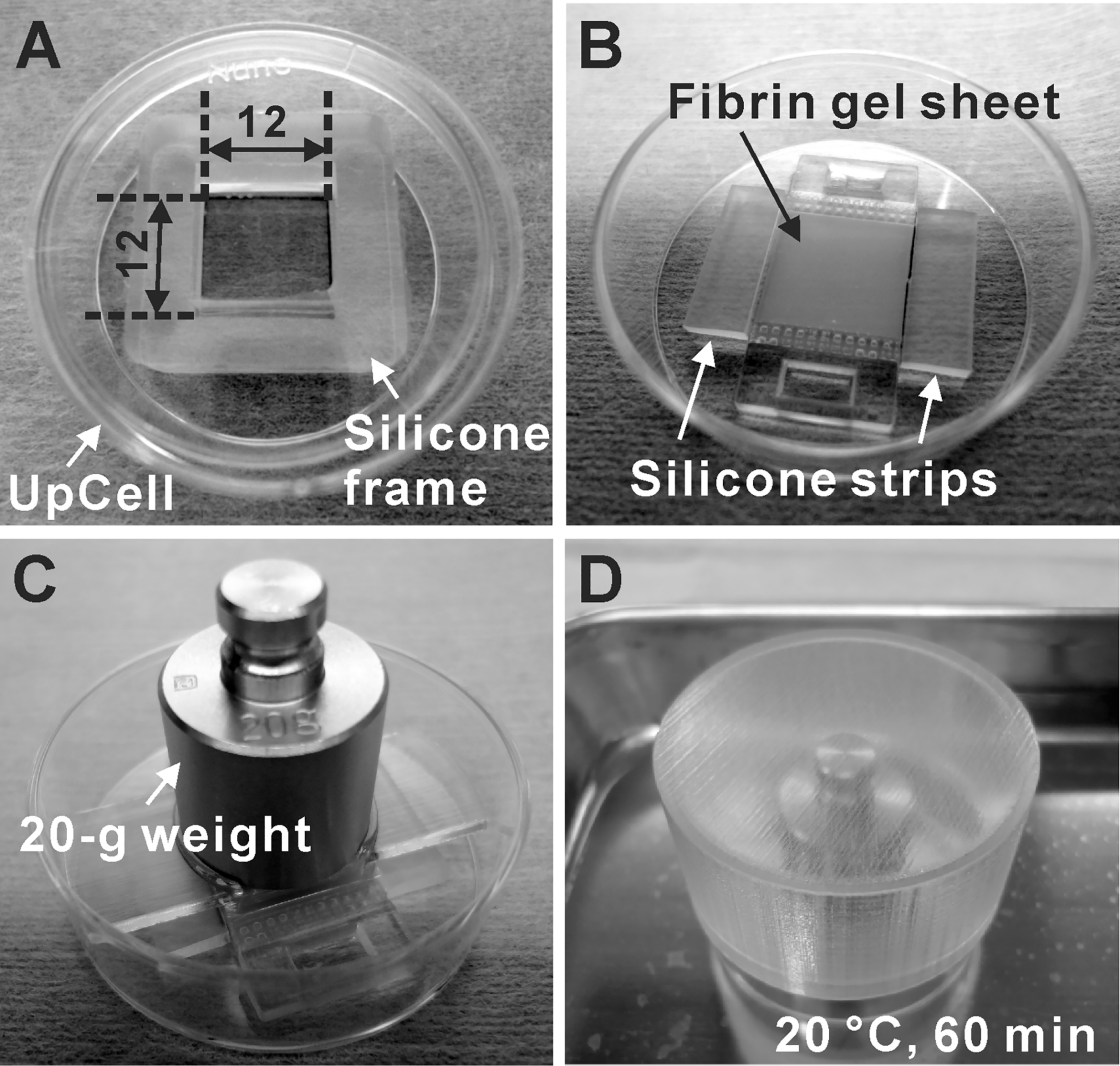
Cardiac cell sheet engineering. (A) Human iPSC-derived cardiomyocytes were cultured on a temperature-responsive dish (UpCell) with a square silicone frame. The unit of measure of the numbers in the figure is mm. (B) Medium and a silicone frame were removed and two silicone strips were put along the facing edges of the cardiac cell sheet. Then the fibrin gel sheet was put on the cardiac cell sheet. (C) A 20-g weight was put on the fibrin gel sheet to attach the fibrin gel sheet closely to the cardiac cell sheet. (D) The dish was covered by a lid made by a 3D printer, and incubated at 20°C for 1hour to transfer the cardiac cell sheet.

Sterilized silicone frames were attached on the surface of temperature-responsive dishes (UpCell; CellSeed, Tokyo, Japan) to restrict the cell culture area to 12-mm square (Fig 2A). The culture surfaces were coated with FBS overnight before cell seeding. On these surfaces, purified iPSC-derived cardiomyocytes were seeded at 3 × 10^5^ cells/cm^2^ (Day 27), and cultured in Medium A in a humidified incubator with 5% CO_2_ at 37°C. The medium was changed to fresh medium on the day after cell seeding, and then every other day. On Day 32 or 33, confluent iPSC-derived cardiomyocytes, i.e. cardiac cell sheets were transferred to the surface of fibrin gel sheets as follows. A few hours before the cell sheet transfer, the medium was changed to Medium B. The high concentration of potassium ions in Medium B attenuated the beating of cardiomyocytes by inducing depolarization, and thus prevented the shrinkage of cardiac cell sheets during the transfer process. The medium and silicone frame were removed, and two silicone strips (15 mm × 5 mm × 2 mm thick) were put along two opposed edges of the cardiac cell sheet (Fig 2B) to prevent the slip of a fibrin gel sheet. The fibrin gel sheet equilibrated with Medium B was put on the cardiac cell sheet (Fig 2B), and a 20-g weight was put on the fibrin gel sheet (Fig 2C). The dish was covered with a lid (Fig 2D) and incubated at 20°C for 1 hour in a humidified low temperature incubator with 5% CO_2_ (WAKEN B TECH, Kyoto, Japan). This procedure allowed the cardiac cell sheet to detach from the temperature-responsive surface and attach to the surface of the fibrin gel sheet. After the cell sheet transfer, the cardiac cell sheet with the fibrin gel sheet (cardiac cell sheet-tissue) was put in a dish with the cell sheet side up, and incubated in Medium B for additional 1 hour at 20°C to firmly attach the cell sheet on the fibrin gel sheet without beating. Then the medium was changed to Medium C (defined as Medium 199 (Catalog Number 12340; Thermo Fisher Scientific) containing 10% FBS, 500 KIU/ml aprotinin, and Penicillin-Streptomycin) and the cardiac cell sheet was cultured in a humidified incubator with 5% CO_2_ at 37°C. The medium was changed to fresh medium every 2–3 days. The contractile force measurement of the cardiac cell sheets were conducted from 4 to 10 days (Day 36–43) after the cell sheet transfer.

### Microscopic and macroscopic observation

The phase-contrast microscopic images and movies of iPSC-derived cardiomyocytes on a temperature-responsive dish and on a fibrin gel sheet were obtained by an inverted microscope (ECLIPSE TE300; Nikon, Tokyo, Japan) equipped with a 3CCD digital camera (HV-D28s; Nikon) and video cassette recorder (WV-DR9; SONY, Tokyo, Japan). The macroscopic movies of cardiac cell sheets on fibrin gel sheets were obtained by a digital microscope (VHX-900; KEYENCE, Osaka, Japan).

To observe intracellular structure of cardiac cell sheet-tissues, they were fixed with 4% paraformaldehyde for 15 minutes, permeabilized and blocked with 0.15% Triton X-100 and 2% bovine serum albumin (BSA) for 20 minutes, and stained with 0.33 μM Alexa Fluor 488 Phalloidin (Thermo Fisher Scientific) for 6 hours. They were mounted on coverslips with anti-fade solution (ProLong Gold Antifade Reagent with DAPI; Thermo Fisher Scientific) and observed by confocal fluorescence microscopy (FV1200; Olympus, Tokyo, Japan).

To observe intracellular Ca^2+^ transient of cardiac cell sheet-tissues, they were stained with 5 μM Fluo-8 (AAT Bioquest, Sunnyvale, CA, USA) in Medium C for 1 hour, and immersed in phenol red-free DMEM (Catalog Number 08489–45; Nacalai Tesque) containing 10% FBS, 500 KIU/ml aprotinin, 30 mM 2,3-Butanedione monoxime (Sigma-Aldrich), and Penicillin-Streptomycin. Fluorescence change due to the intracellular Ca^2+^ transient was obtained by a tandem-lens macroscope (Brainvision, Tokyo, Japan) equipped with a 100 × 100 pixels CMOS fast camera system (MiCAM ULTIMA-L; Brainvision), and processed by image processing softwares (BV_Ana, BV_Workbench; Brainvision) for baseline correction and colored visualization.

To analyze the thickness of cardiac cell sheets on fibrin gel sheets, they were fixed with 4% paraformaldehyde, immersed in saline, and the cross-sectional images were obtained by an optical coherence microscopy (OCM) system (Panasonic, Osaka, Japan) [31]. The cross-sectional area and thickness of cardiac cell sheets were analyzed using a graphic design software (Corel DRAW; Corel, Ottawa, Canada) and an image processing software (ImageJ; National Institute of Health, Bethesda, MD, USA).

From some cardiac cell sheet-tissues, hematoxylin and eosin (H&E) stained paraffin sections were also prepared and the microscopic images were obtained by a microscope (ECLIPSE E800; Nikon) equipped with a digital camera (DS-Ri1; Nikon).

### Contractile force measurement system

**Fig 3:**
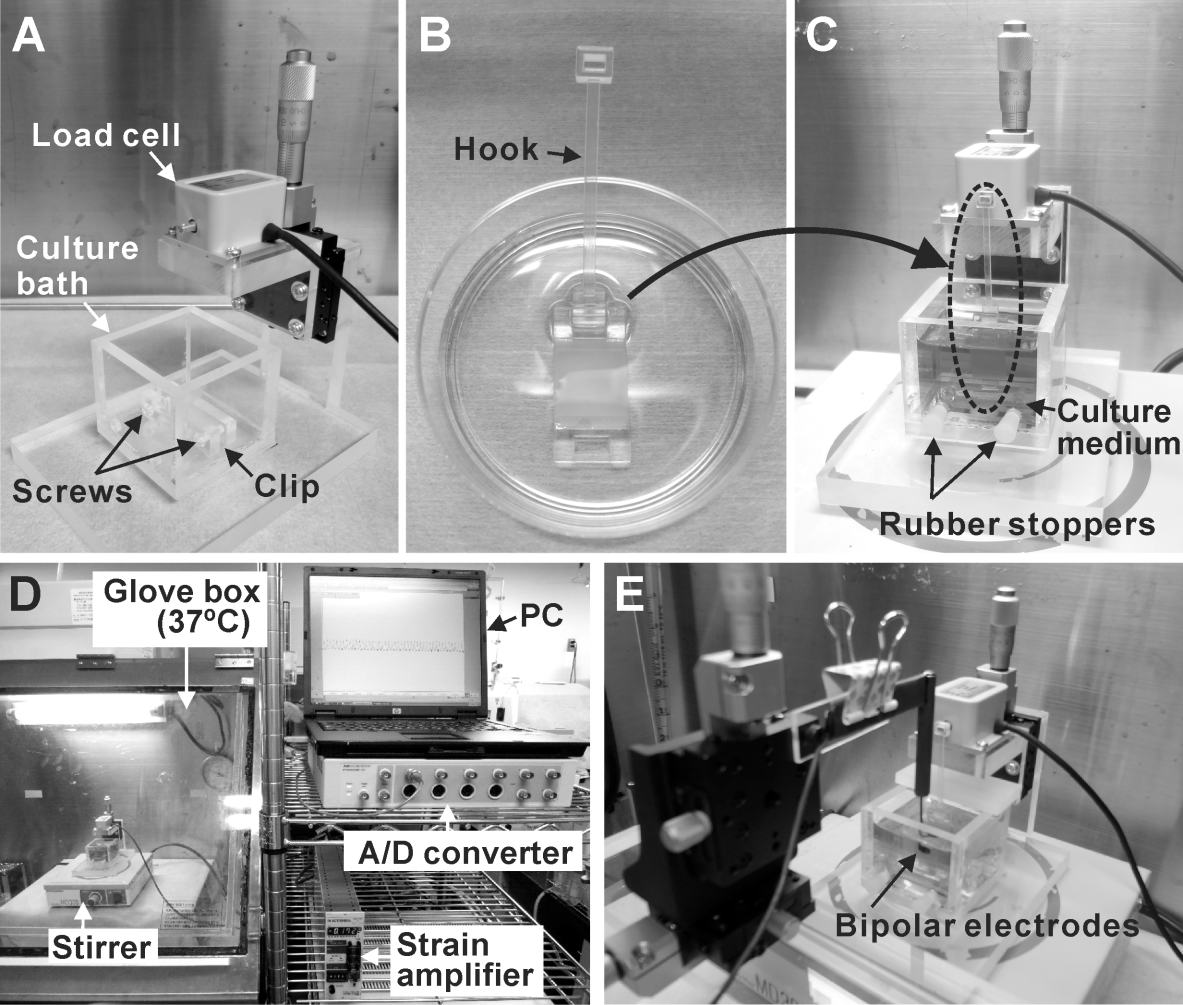
Configuration of contractile force measurement system. (A) The appearance of a contractile force measurement device. (B) The hook made by a 3D printer was fixed to the handle. (C) The cardiac cell sheet-tissue was mounted to the force measurement device vertically and fresh medium (Medium D) was poured. (D) The entire appearance of a contractile force measurement system. (E) The appearance of electrical pacing system.

As shown in Fig 3A, the contractile force measurement device is composed of a load cell (LVS-10GA; Kyowa Electronic Instruments, Tokyo, Japan) and a culture bath made of acrylic plates. On the bottom of the culture bath, a clip is fixed to hold the handle of a fibrin gel sheet. There are two holes at the front of the culture bath, to allow a screwdriver to tighten the screws of the clip. The height of a load cell can be adjusted by a uniaxial stage (Chuo Precision Industrial, Tokyo, Japan). The fibrin gel sheet was hung from a sensor rod of the load cell by using a hook made by a 3D printer (Fig 3B and 3C), and the lower handle of the fibrin gel sheet was held by the clip on the bottom of the culture bath. The holes at the front of the culture bath were closed with rubber stoppers, and 40 ml Medium D (defined as Medium 199, Hank’s (Catalog Number 12350; Thermo Fisher Scientific) containing 10% FBS, 500 KIU/ml aprotinin, and Penicillin-Streptomycin) was poured into the bath. Because the Medium D consists of Hanks’ salts, it does not require CO_2_ to maintain physiological pH. A small magnetic bar was put in the culture bath, and the medium was stirred gently during the measurement. The contractile force measurement was conducted at 37°C in a glove box (Fig 3D). The load cell was connected to a strain amplifier (DPM-712B; Kyowa Electronic Instruments), and the contractile force was recorded by a personal computer through an A/D converter (Power Lab 8/30; ADInstruments, Bella Vista, Australia) (Fig 3D). The culture baths, hooks, magnetic bars, and rubber stoppers were sterilized with ethylene oxide gas before used.

It should be noted here that the results of contractile force measurement experiments were analyzed from the data including multiple data of cardiac cell sheet-tissues prepared from a same differentiation batch, and data from different differentiation batches in some experiments.

In some cases, electrical pacing of the cardiac cell sheet was conducted using bipolar platinum electrodes. The distance between the bipolar electrodes was 3 mm. The electrodes were positioned above the center of the cardiac cell sheet, and the distance between the electrode and the cardiac cell sheet was adjusted to about 100 μm (Fig 3E). Biphasic pacing pulses (2.3–3.8 V, 20-msec pulse duration, 60–200 paces per minutes (ppm)) were applied by an electrical stimulator (UPS-801; Unique Medical, Tokyo, Japan). The pacing voltage was determined as 1.5 times the minimum voltage to activate the contraction of a cardiac cell sheet.

To conduct drug testing, adrenaline was administered to some cardiac cell sheet tissues. During the contractile force measurement, 36.6 μl of 5.46 mM adrenaline stock solution (Bosmin Injection; Daiichi Sankyo, Tokyo, Japan) was added to the culture medium (40 ml) in a culture bath, resulting in a final concentration of 5 μM.

## Results

### Preparation of cardiac cell sheet-tissue for contractile force measurement

**Fig 4:**
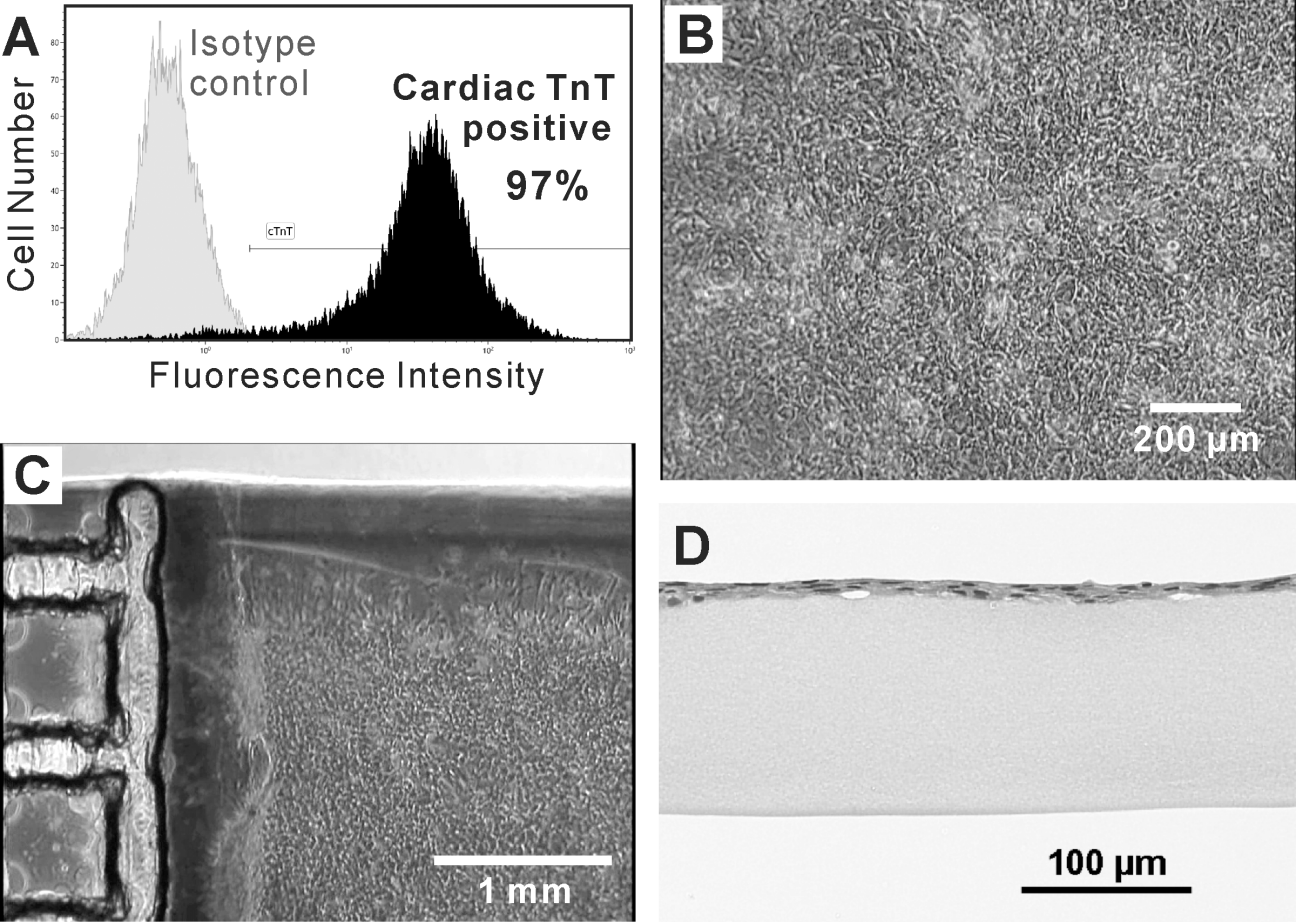
Flow cytometry and microscopic observation of cardiac cell sheets. (A) A histogram of fluorescently labeled human iPSC-derived cells after twice puromycin treatments, analyzed by flow cytometry. The purity of cardiomyocytes (cardiac TnT-positive cells) was confirmed. (B) A phase-contrast microscopic image of a cardiac cell sheet cultured on a temperature-responsive dish at 7 days after cell seeding. (C) A phase-contrast microscopic image of a cardiac cell sheet transferred on a fibrin gel sheet at 3 days after cell sheet transfer. (D) A microscopic image of H&E stained paraffin section of a cardiac cell sheet on a fibrin gel sheet after cell sheet transfer.

**Fig 5:**
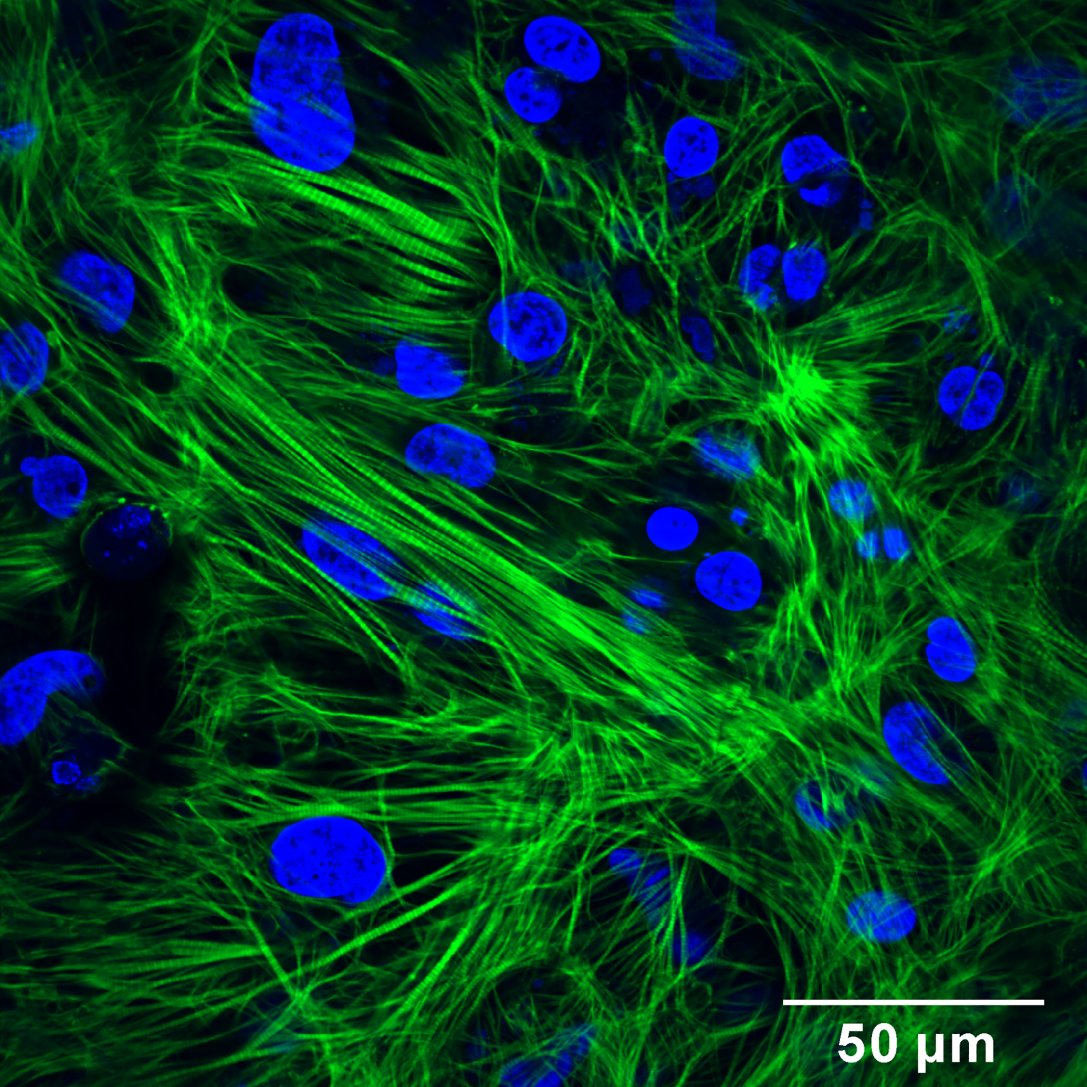
Confocal fluorescence microscopy of cardiac cell sheets. The cardiac cell sheet-tissue was fixed with 4% paraformaldehyde at 7 days after cell sheet transfer. Actin filaments (green) and nuclei (blue) were stained fluorescently.

After two puromycin treatment processes, the percentage of human iPSC-derived cardiomyocytes was 95.2 ± 5.6% (mean ± SD of 4 independent experiments), analyzed by flow cytometry (Fig 4A). These purified cardiomyocytes were seeded on the temperature-responsive surfaces and reached confluency in 4–6 days, connecting to each other to form cardiac cell sheets (Fig 4B). The cardiomyocytes beat synchronously and periodically on the surfaces (S1 Movie). During the cell sheet transfer procedure, the beating of cardiac cell sheets was stopped by adding high concentration of potassium ion. Otherwise, cardiac cell sheets could shrink on the surface of a fibrin gel sheet immediately after the transfer. After the cell sheet transfer procedure, we confirmed that the cardiac cell sheet was attached on the surface of a fibrin gel sheet by phase-contrast microscopy (Fig 4C) and H&E staining of paraffin sections (Fig 4D). The beating of cardiac cell sheets recovered immediately after the medium was changed to Medium C. The beating amplitude gradually increased in several days, and could be observed with the naked eye (S2 and S3 Movies). A confocal fluorescence microscopic image of actin filament in the cardiac cell sheet-tissue revealed that striated sarcomeric structures were formed over the wide range of cell sheet (Fig 5). The observation of intracellular Ca^2+^ transient revealed highly synchronized Ca^2+^ transients over the whole range of the cardiac cell sheet-tissue (S4 Movie), which indicated substantial intercellular connections.

### Contractile force measurement of cardiac cell sheet-tissue

**Fig 6:**
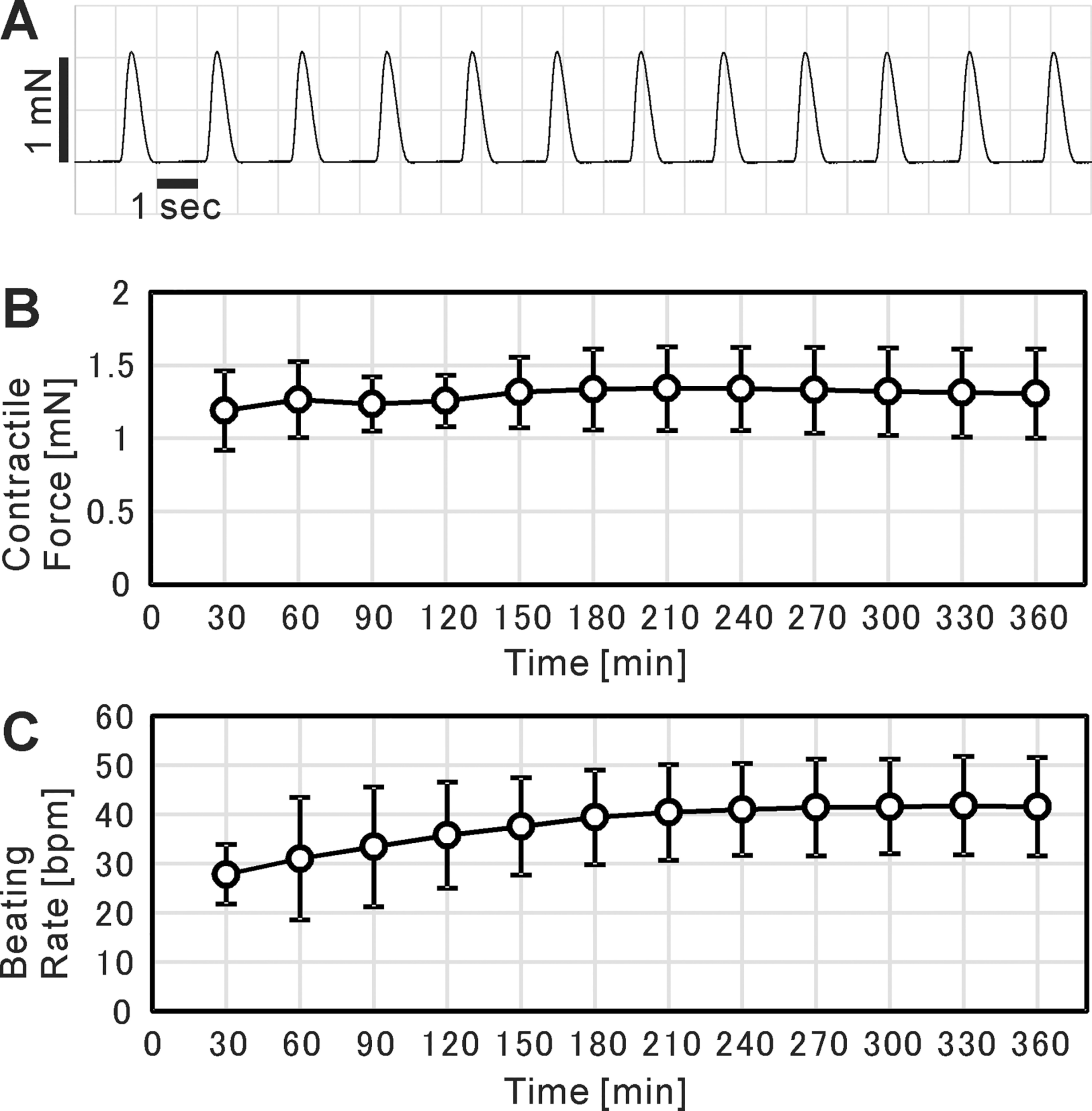
Contractile force measurement of cardiac cell sheet-tissues. (A) A representative contractile force trace of a cardiac cell sheet-tissue. (B, C) Time course analysis of contractile forces (B) and beating rates (C) of cardiac cell sheet-tissues for 6 hours. Each value of contractile forces at a certain time point was determined as the mean of those values in one minute just before that time point. Each value of beating rates at a certain time point was determined as the number of beatings in one minute just before that time point. Results are presented as mean ± SD for 5 cardiac cell sheet-tissues. Among the 5 cardiac cell sheet-tissues, 2 samples were prepared from a same differentiation batch. The other 3 samples were prepared from different 3 differentiation batches respectively.

The cardiac cell sheet-tissue was mounted to the force measurement device and very clear contractile force profiles around 1 mN was detected (Fig 6A). We could measure the stable contractile forces for at least 6 hours (Fig 6B). The beating rate tended to increase at the start of measurement and became stable in a few hours (Fig 6C).

**Fig 7:**
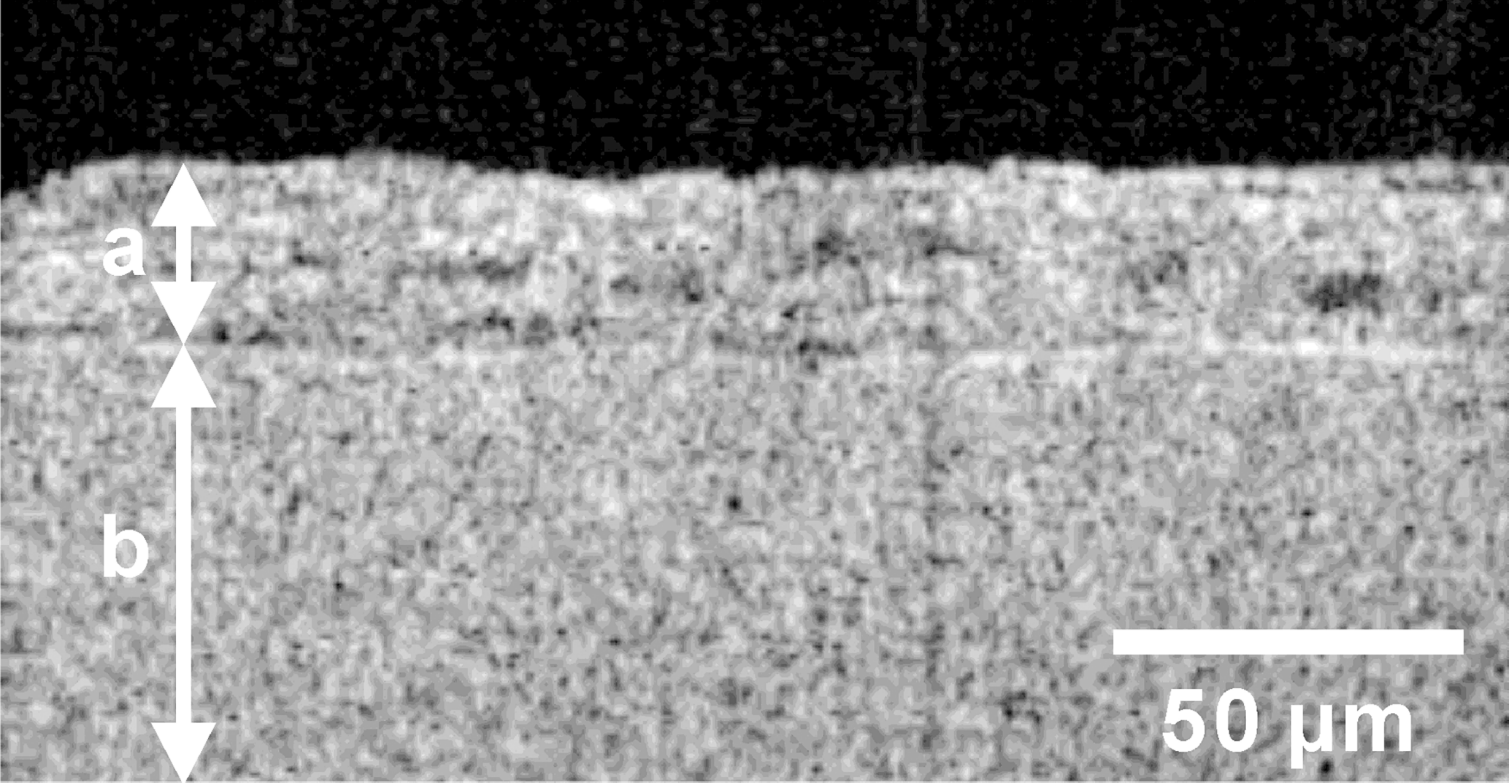
A representative cross-sectional image of a cardiac cell sheet-tissue obtained by OCM system. The bidirectional arrow (a) indicates the layer of a cardiac cell sheet and the bidirectional arrow (b) indicates the layer of a fibrin gel sheet.

Then we analyzed the value of a contractile force per cross-sectional area of a cardiac cell sheet. The contractile force was obtained at 1 hour from the start of measurement. Then the thickness of that cardiac cell sheet was analyzed by OCM (Fig 7). As a result, the contractile force was 0.85 ± 0.11 mN, and the thickness was 21.5 ± 1.7 μm (mean ± SD of 4 cardiac cell sheets). Because the width of cardiac cell sheets was 12 mm, the contractile force per cross-sectional area was calculated to be 3.3 ± 0.6 mN/mm^2^ (mean ± SD of 4 cardiac cell sheets).

**Fig 8:**
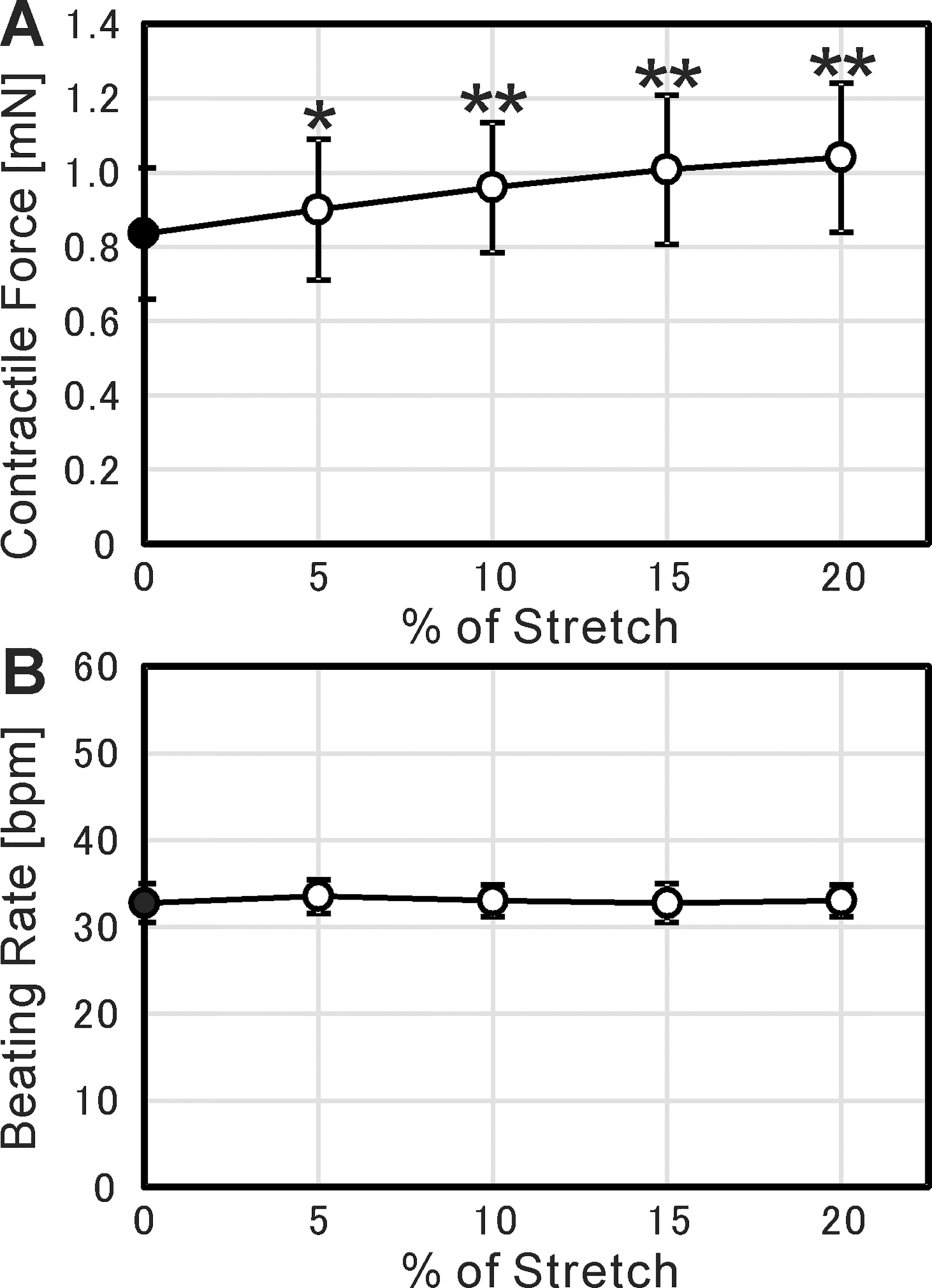
Frank-Starling mechanism of cardiac cell sheet-tissues. (A) Relationship between contractile force and % of stretch. Each value of contractile forces was determined as the mean of those values in one minute. (B) Relationship between beating rate and % of stretch. Each value of beating rates was determined as the mean of those values in one minute. In both figures, the results are presented as mean ± SD for 4 cardiac cell sheet-tissues, prepared from a same differentiation batch. Each value of contractile forces and beating rates was statistically compared to the value at 0% stretch (closed circles) using Student’s t-test (* p < 0.05, ** p < 0.01).

We also analyzed the dependence of contractile force on the stretch of cardiac cell sheet-tissues. We stretched the cardiac cell sheet-tissues by 5% up to 20% at 15-minute intervals and measure the contractile force. As a result, we confirmed a positive relationship between them (Fig 8A). This positive relationship is physiologically relevant to actual cardiac tissue, known as Frank-Starling mechanism [32]. On the other hand, the beating rate was constant regardless of the stretch (Fig 8B).

### Force-frequency relationship and frequency dependent acceleration of relaxation

**Fig 9:**
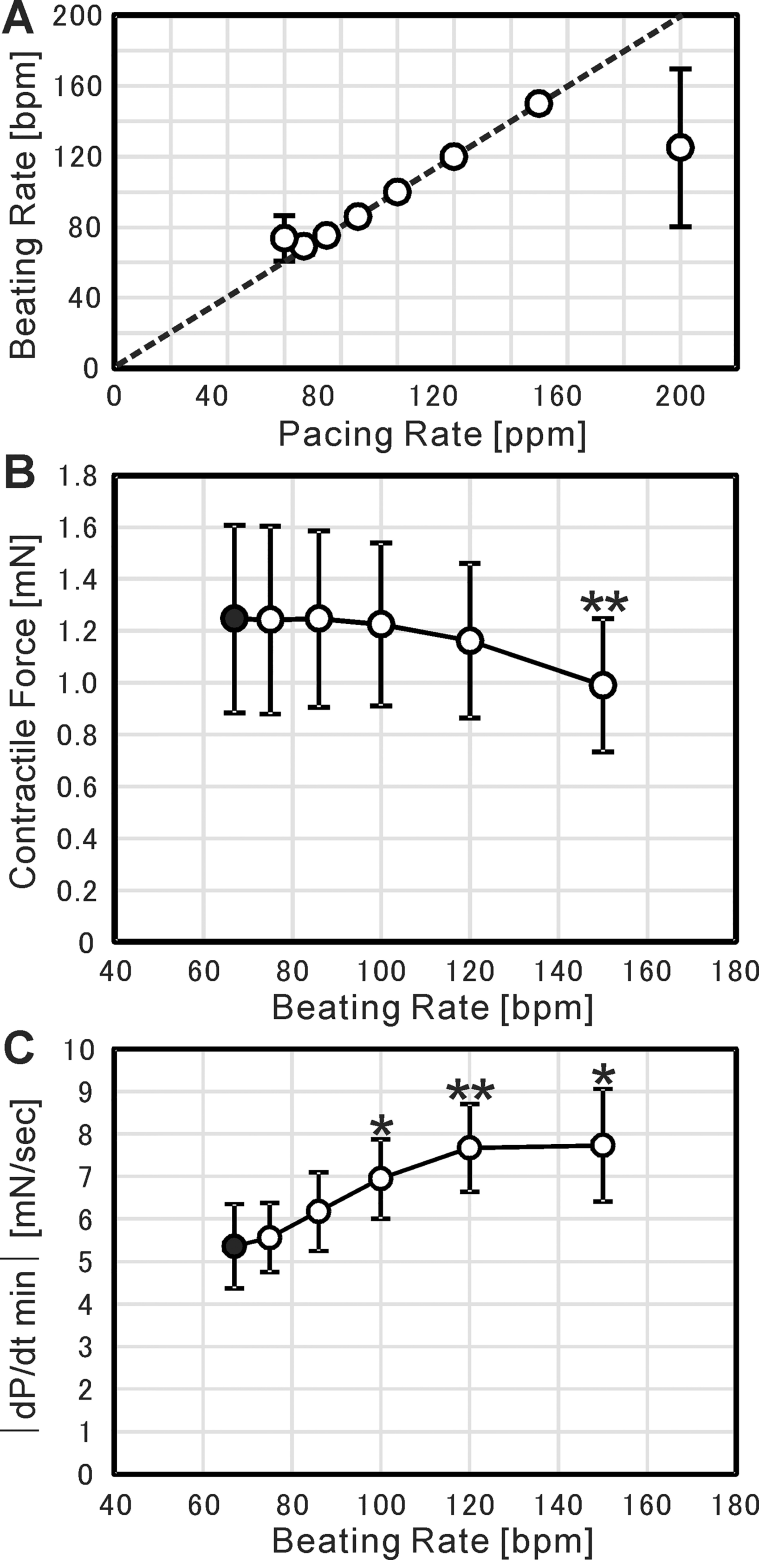
Contractile force measurement under electrical pacing. (A) Relationship between pacing rate and beating rate. Each value of beating rates was determined as the number of beatings in one minute during pacing. The dotted line indicates the equality between pacing rate and beating rate. (B) Relationship between beating rate and contractile force (i.e., force-frequency relationship). Each value of contractile forces was determined as the mean of those values in one minute during pacing. (C) Relationship between beating rate and |dP/dt min|. Each value of |dP/dt min| was determined as the mean of those values in one minute during pacing. In all figures, the results are presented as mean ± SD for 5 cardiac cell sheet-tissues. Among the 5 cardiac cell sheet-tissues, 2 samples were prepared from a same differentiation batch. The other 3 samples were prepared from different 3 differentiation batches respectively. For (B) and (C), each value of contractile forces was statistically compared to the value at 67 bpm (closed circles) using Student’s t-test (* p < 0.05, ** p < 0.01).

The force-frequency relationship of cardiac cell sheets was examined by electrical pacing. The electrical pulses were applied with the interval of 1.0, 0.9, 0.8, 0.7, 0.6, 0.5, 0.4, and 0.3 seconds, which corresponded to the pacing rate of 60, 67, 75, 86, 100, 120, 150, and 200 ppm, respectively. Fig 9A shows the relationship between the pacing rate and the beating rate of cardiac cell sheets. The data on the dotted line in Fig 9A indicates that the beating of cardiac cell sheets completely followed the electrical pacing in the range from 67 to 150 ppm. Therefore, the force-frequency relationship was examined at this range as shown in Fig 9B. Compared with the contractile force at 67 bpm, there was no significant difference in the contractile force up to 120 bpm. However, at 150 bpm the contractile force decreased significantly by 20%. The relationship between the pacing rate and the maximum relaxation speed (|dP/dt min|) was also analyzed as shown in Fig 9C. As a result, we confirmed a positive relationship between them. This positive relationship is physiologically relevant to actual cardiac tissue, known as frequency dependent acceleration of relaxation (FDAR) [33].

### Response to adrenaline administration

**Fig 10:**
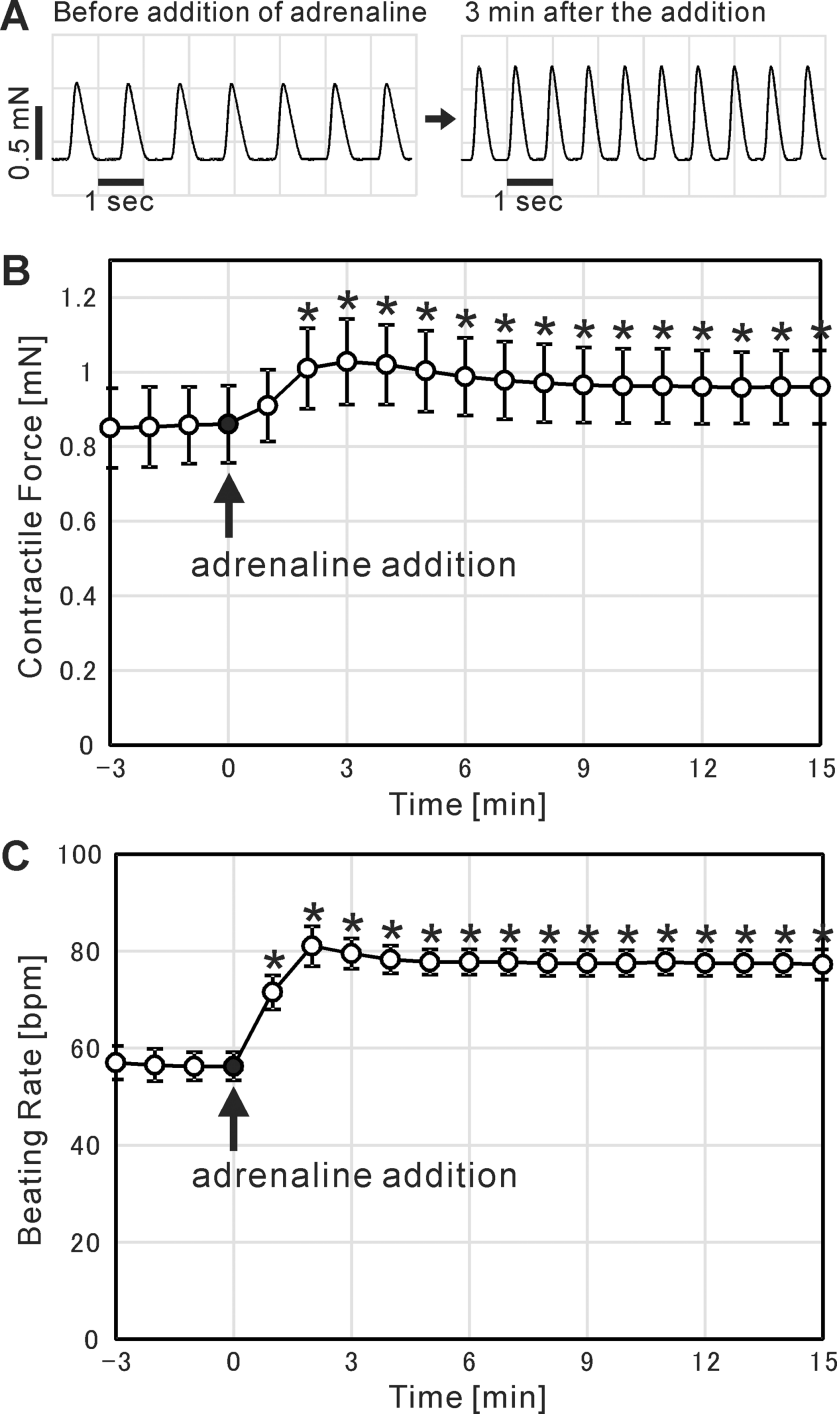
The effect of adrenaline administration on the contractility of cardiac cell sheet-tissues. (A) Representative contractile force traces of a cardiac cell sheet-tissue before and after the administration of 5 μM adrenaline. (B, C) Time course analysis of contractile forces (B) and beating rates (C) of cardiac cell sheet-tissues before and after the administration of 5 μM adrenaline. Each value of contractile forces at a certain time point was determined as the mean of those values in one minute just before that time point. Each value of beating rates at a certain time point was determined as the number of beatings in one minute just before that time point. The results are presented as mean ± SD for 4 cardiac cell sheet-tissues, prepared from a same differentiation batch. Each value of contractile forces and beating rates (open circles) was statistically compared to the value at the time point of adrenaline addition (closed circles) respectively using Student’s t-test (* p < 0.01).

The effect of adrenaline administration on the contractile force and beating rate of cardiac cell sheets was examined. By the addition of 5 μM adrenaline, both the contractile force and the beating rate significantly increased within a few minutes (Fig 10A–10C). The contractile force became maximum at 3 minutes after the adrenaline addition, increased 1.20 ± 0.03 times that before the addition (mean ± SD for 4 cardiac cell sheets). The beating rate increased 1.44 ± 0.04 times that before the addition in two minutes (mean ± SD for 4 cardiac cell sheets).

## Discussion

In order to evaluate the contractile properties of engineered cardiac cell sheet-tissues with measurable quantities, in the present study we developed a system to measure the contractile forces of cardiac cell sheet-tissues. The cardiac cell sheet-tissues were made of human iPSC-derived cardiomyocytes.

Fibrin gel sheets with handles made by a 3D printer were utilized to connect the cardiac cell sheet-tissues to the force measurement device (Fig 1). The cardiac cell sheets were transferred from temperature-responsive dishes to the fibrin gel sheets by a cell sheet transfer technique developed previously (Fig 2) [7, 8]. The cardiac cell sheet-tissue with a fibrin gel sheet was mounted to the load cell vertically (Fig 3C). We found that the vertical rather than horizontal mounting of cardiac cell sheet-tissues significantly reduced the noise of force measurement (S1 Fig). We used culture medium with Hanks’ salts for force measurement so that we could maintain the physiological pH without controlling environmental CO_2_ concentration, simplifying the system. As a result, we achieved clear and stable beating for more than 6 hours and successfully measured contractile force (Fig 6).

The mean contractile force per cross-sectional area was 3.3 mN/mm^2^ which is higher than the many reported values of engineered cardiac tissues derived from human pluripotent stem cells (ESCs and iPSCs): 0.08 mN/mm^2^ [26], 0.12 mN/mm^2^ [24], 0.57 mN/mm^2^ [25], 0.62 mN/ mm^2^ [27], and 1.34 mN/mm^2^ [28]. However, some recent studies succeeded to create more powerful engineered cardiac tissues: 6.2 mN/mm^2^ [34], 11.8 mN/mm^2^ [29], and 23.2 mN/mm^2^ [35]. Furthermore, the contractile forces of human adult cardiac tissues were reported to be 40–50 mN/mm^2^ [36, 37], which is considerably higher than those of engineered cardiac tissues. Some reasons for this difference can be assumed as follows.

One is the immaturity of pluripotent stem cell-derived cardiomyocytes in cardiac cell sheet-tissues. The fact that the contractile force is higher than the many reported values listed in the discussion, and formation of sarcomeric structure shown in Fig 7, indicate that the maturation have proceeded to some degree. The relationship between contractile force and beating rate (Force-Frequency Relationship: FFR) is also one index of cardiac maturation. The FFR in human mature cardiac tissues was reported to be positive [38, 39]. On the other hand, many studies reported that the FFR of pluripotent stem cell-derived cardiac tissues was negative, which is considered to be due to their immaturity [25, 28, 34, 40]. In the present study, although the contractile force maintained the same level at a beating rate below 120 bpm, it decreased significantly at 150 bpm (Fig 9B). The result indicates that the maturation of our iPSC-derived cardiac cell sheet-tissues is still insufficient. Although the FFR was negative, we confirmed the Frank-Starling mechanism (Fig 8A) and the FDAR (Fig 9C), which are relevant to actual maturated cardiac tissue. It was reported that the maturation of pluripotent stem cell-derived cardiac tissues could be induced by a long-term culture period [41, 42], administration of biochemical substances [34, 43, 44], and long-term rapid pacing [28, 45]. These conditions should also be examined in the present experimental setup.

Another possible reason for the lower contractile forces is the structural difference between the cardiac cell sheet-tissues and adult cardiac tissues. The cardiomyocytes in adult cardiac tissues are highly aligned along their beating directions and are composed as multicellular muscle fiber structures. On the other hand, we could not find the cell alignment in the cardiac cell sheet-tissues by confocal fluorescence microscopy (Fig 5). The beating directions of cardiomyocytes in the cardiac cell sheet-tissues are also random (S1 Movie). The lack of cell alignment leads to the underestimation of contractile force, and may prevent the functional maturation of cardiac tissue. There are plenty of studies showing that cell alignment in engineered cardiac tissues improve their contractile properties [46–50]. We also originally developed the methods to fabricate cell sheets with cell alignment, using microtextured temperature-responsive substrates [51], microcontact printing of fibronectin on temperature-responsive surface [52], and micropatterned temperature-responsive surface [53]. Using these technologies, we are planning to investigate the effect of cell alignment on the contractile properties of cardiac cell sheet-tissues.

Another difference between the cardiac cell sheet-tissues and human adult cardiac tissues is the presence of non-cardiomyocyte cells. Major non-cardiomyocyte cells comprising intact cardiac tissues are cardiac fibroblasts, vascular endothelial cells, and vascular smooth muscle cells. There are several studies showing that co-culture of these non-cardiomyocyte cells induces functional maturation of cardiomyocytes and engineered cardiac tissues [27, 49, 54–56], possibly due to the direct cell-cell interactions and paracrine effects. Additionally, the presence of vascular cells is also important for better engraftment of engineered cardiac tissues after transplantation. Our previous studies demonstrated that the presence of vascular cells in cell sheet-tissues significantly improved their engraftment to the host tissues by promoting vascularization [57–59]. Furthermore, to create thick cardiac cell sheet-tissues (e.g., > 100 μm in thickness) *in vitro* without necrosis, microvascular networks must be constructed in the tissues for nutrient supply [20, 21]. On the other hand, too many non-cardiomyocyte cells in the tissues can decrease the contractility and possibly cause arrhythmia by disturbing action potential propagation [60]. Considering the above conditions, the composition of engineered cardiac tissues must be optimized to realize as powerful a contractility as possible.

The effect of cardiac subtypes on the contractile force also should be considered. We analyzed the percentage of ventricular myosin light chain-2 (MLC2v) positive cardiomyocytes by flow cytometry (S2 Fig). We confirmed that the percentage of MLC2v positive cardiomyocytes was 99.0%, indicating that almost all cardiomyocytes were destined to become ventricular cardiomyocytes. As for the effect of cardiac subtypes on the contractile force, there are several reports comparing the contractile forces of atrium and ventricle. However, the results of them were inconsistent. Ruf et al. [61] and Piroddi et al. [62] reported that the contractile force per cross sectional area of atrium is higher than that of ventricle. On the other hand, Morano et al. [63] reported the opposite result. Considering that the contractile function of heart is mainly determined by ventricle, we consider ventricular cardiomyocytes preferable as transplants for cardiac regenerative therapy, and also for drug testing. Of course the differentiation methods to prepare ventricular cardiomyocytes and atrium cardiomyocytes separately should be established in the future for more precise analysis.

In the present study we also conducted drug testing of the iPSC-derived cardiac cell sheet-tissues by adding adrenaline. As a result, both contractile force and beating rate were increased in the same way as actual cardiac tissues (Fig 10). The result indicates that the present system can be also utilized for drug screening in drug development. For the same purpose, there are some existing analyzing systems to quantify the contractility of cardiomyocytes. xCELLigence (ACEA Biosciences, San Diego, CA, USA) and Cell Motion Imaging System (SONY Biotechnology, San Jose, CA, USA) can evaluate the contractility of cardiomyocytes by comprehensively detecting the microscopic movement of cardiomyocytes on cell culture plates. There are several studies conducting drug testing of human pluripotent stem cell-derived cardiomyocytes by utilizing these systems [64–67]. Although these systems are excellent in throughput performance, there might be still room for investigating whether the microscopic movement of cardiomyocytes on the dishes completely corresponds to the contractility of cardiac tissues. Lind et al. recently developed a further sophisticated system [68]. They cultured laminar cardiac tissues on flexible substrates incorporating strain gauge wires, and quantified the contractility by electrically detecting the deflection of the substrates due to the cardiac contraction. Compared to the above systems, the system developed in the present study might be inferior in throughput performance. However, we consider there are still some advantages of the present system. First, the absolute value of contractile force is so large that the data is sufficiently clear (Fig 6A), which can allow the detailed quantification of contractile properties. Second, the system basically follows the conventional experimental setup in muscle physiology measuring isometric contractile force, so that the interpretation of the data is simple and reliable.

As for the throughput performance, we consider that it is possible to improve it satisfactory by simplifying the preparation of cardiac cell sheet-tissues and their connection to the device. From the requirement of evaluating the contractility of cardiac cell sheet-tissues as a transplant for regenerative therapies, it is indispensable to measure the contractile force of cardiac cell sheet-tissues prepared by using temperature-responsive dishes. On the other hand, for the purpose of the application to drug testing, more simple method to prepare cardiac cell sheet tissues can be considered including direct cell seeding. As for the cost per data point, the cost of preparing or purchasing iPSC-derived cardiomyocytes might be a major running cost. In the case of our method, the size of a cardiac cell sheet is 144 mm^2^ (12 mm × 12 mm). In the case of xCELLigence, which might be the most high-throughput system at present, 96-well plates were used and the area of each well is 32 mm^2^. Therefore, the running cost per data point of our method is about 4.5 times higher than that of xCELLigence. However, this running cost can be reduced by optimizing the size and cell density of the cardiac cell sheet-tissue, and by improving the culture conditions to induce maturation.

In conclusion, the contractile force measurement system developed in the present study is useful for the evaluation of contractile properties of engineered cardiac cell sheet-tissues, and for *in vitro* cardiac drug testing. Additionally, if the cardiac cell sheet-tissues are created from iPSCs with gene mutations causing cardiomyopathy, the present system will be also useful for pathological investigation.

## Supporting information

S1 Fig **Horizontal type contractile force measurement device (A) and contractile force trace (B)**.
(PDF)

S2 Fig Flow cytometrical analysis of ventricular myosin light chain-2 (MLC2v) positive cardiomyocytes. (PDF)

S1 Movie Phase-contrast microscopic view of a cardiac cell sheet cultured on a temperature-responsive dish. The movie was recorded after 7 days from cell seeding.
(WMV)

S2 Movie Phase-contrast microscopic view of a cardiac cell sheet-tissue with a fibrin gel sheet. The movie was recorded after 3 days from cell sheet transfer procedure.
(WMV)

S3 Movie Macroscopic view of a cardiac cell sheet-tissue. The movie was recorded after 8 days from cell sheet transfer procedure.
(WMV)

S4 Movie Macroscopic view of intracellular Ca^2+^ transients in a cardiac cell sheet-tissue. The movie was recorded after 4 days from cell sheet transfer procedure.
(WMV)
