# Supplementary material for: Contractile force measurement of human induced pluripotent stem cell-derived cardiac cell sheet-tissue
Source: PLoS One. 2018 May 23;13(5):e0198026. doi: 10.1371/journal.pone.0198026 (PMC5965888; doi:10.1371/journal.pone.0198026)
Supplement: S1 Fig — Horizontal type contractile force measurement device (A) and contractile force trace (B). (PDF) [file pone.0198026.s001.pdf]

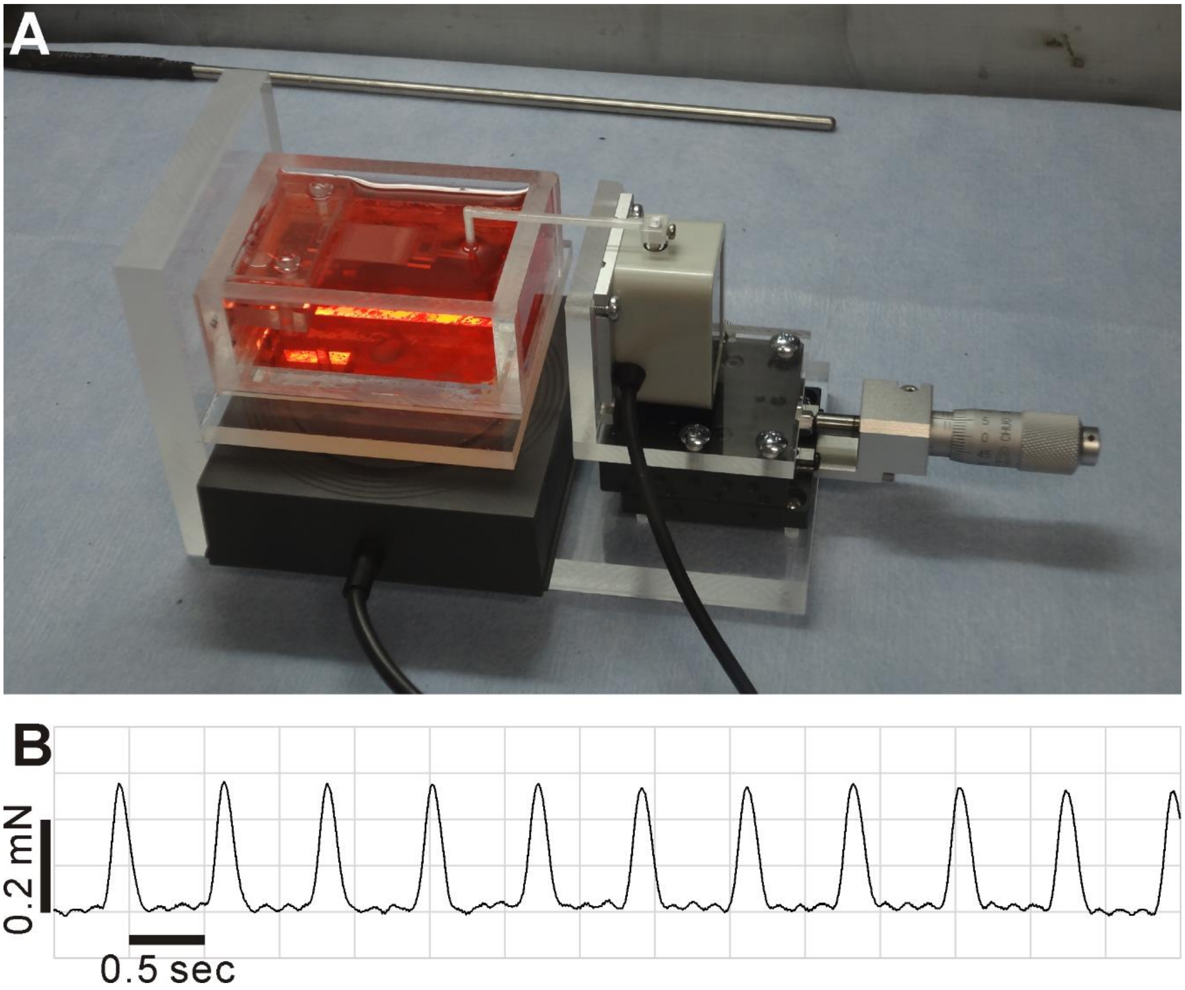

S1 Fig. Horizontal type contractile force measurement device (A) and contractile force trace (B). It should be noted that there is a noise in the contractile force trace due to the vertical shaking of the cardiac cell sheet-tissue. The cardiac cell sheet-tissue was prepared from cardiomyocytes isolated from Sprague-Dawley neonatal rat ventricles according to the method described previously [20]. The animal experiments were performed according to the "Guidelines of Tokyo Women's Medical University on Animal Use" under the approval of institutional ethical committee (approval number: 13-63).
