# Supplementary material for: Contractile force measurement of human induced pluripotent stem cell-derived cardiac cell sheet-tissue
Source: PLoS One. 2018 May 23;13(5):e0198026. doi: 10.1371/journal.pone.0198026 (PMC5965888; doi:10.1371/journal.pone.0198026)
Supplement: S2 Fig — (PDF) [file pone.0198026.s002.pdf]

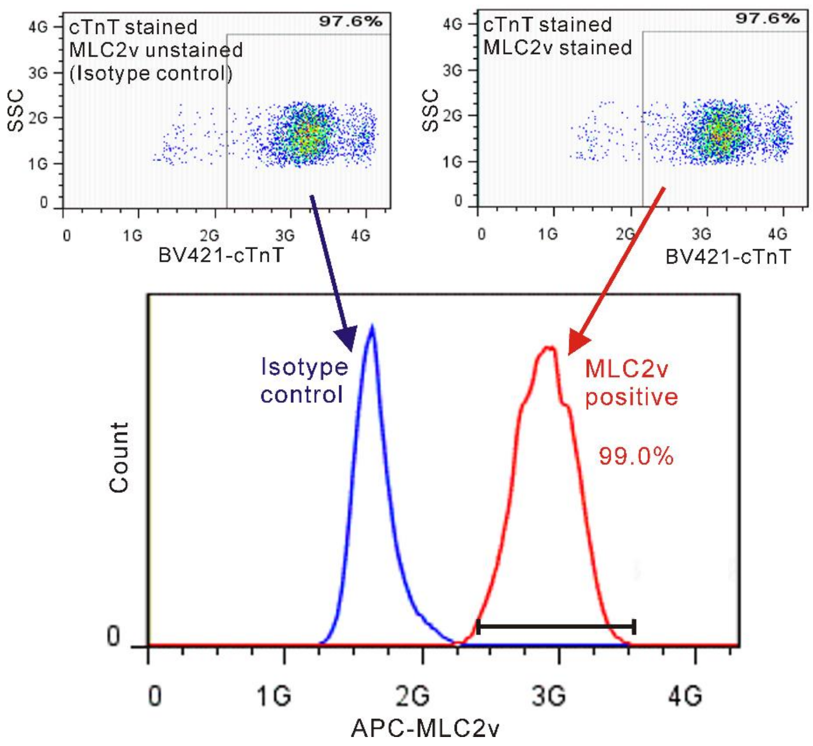

S2 Fig. Flow cytometrical analysis of ventricular myosin light chain-2 (MLC2v) positive cardiomyocytes. After twice puromycin treatments, human iPS-derived cells were fixed with 4% paraformaldehyde for 10 minutes, and labeled with BV421 conjugated cardiac troponin t (cTnT) antibody (Catalog Number 565618; BD Biosciences, San Jose, CA, USA) and Allophycocyanin (APC) conjugated ventricular myosin light chain-2 (MLC2v) antibody (Catalog Number NBP1-30249APC; Novus Biologicals, Littleton, CO, USA) in PBS containing 0.5% saponin and 2% FBS for 2 hours. As an isotype control, APC conjugated recombinant human IgG1 (Catalog Number 130-104-615; Miltenyi Biotec, Bergisch Gladbach, Germany) was used instead of APC conjugated MLC2v antibody. The percentage of MLC2v positive cells in the cTnT positive cells (iPS-derived cardiomyocytes) was analyzed by MoFLo XDP (Beckman Coulter, Brea, CA, USA) and FlowJo software (Treestar, San carlos, CA, USA).
